# Age-stratified diagnostic performance of the ovarian-adnexal reporting and data system for adnexal masses: a focus on school-age children, early, and middle adolescents

**DOI:** 10.3389/fped.2026.1867445

**Published:** 2026-07-15

**Authors:** Rongsen Zhang, Yan Tan, Xiaoye Zhang, Taiqing Zheng, Minghui Liu, Guotao Wang

**Affiliations:** 1 Department of Ultrasound Diagnosis, The Second Xiangya Hospital, Central South University, Changsha, China; 2 Department of Pathology, Hunan Children's Hospital, Changsha, China.

**Keywords:** adnexal masses, child, O-RADS, ultrasonography, adolescent

## Abstract

**Objective:**

To evaluate the age-stratified diagnostic performance of the Ovarian-Adnexal Reporting and Data System (O-RADS) for distinguishing malignant adnexal masses in school-age children, early adolescents, and middle adolescents.

**Methods:**

This retrospective study included 501 pediatric patients with adnexal masses. All masses were classified according to the O-RADS ultrasound lexicon. Diagnostic performance was assessed across three age groups: school-age children, early adolescents, and middle adolescents.

**Results:**

Among 501 masses, 72 were in school-age children, 163 in early adolescents, and 266 in middle adolescents. In school-age children, sensitivity and negative predictive value (NPV) were both 100.0%, specificity 93.6%, and area under the curve (AUC) 0.968; in early adolescents, sensitivity 95.8%, specificity 88.5%, and AUC 0.922; in middle adolescents, sensitivity decreased to 88.2%, specificity to 85.6%, and AUC to 0.869. Positive predictive value (PPV) declined from 71.4% in school-age children to 29.4% in middle adolescents. A significant difference in AUC was observed between middle adolescents and school-age children (*P* = 0.027).

**Conclusion:**

The O-RADS ultrasound demonstrated favorable diagnostic performance in school-age children and early adolescents. The declining performance in middle adolescents, particularly the marked drop in PPV and significant decrease in AUC, underscores the need for age-specific considerations when applying O-RADS in pediatric populations.

## Introduction

Adnexal masses are rare in the pediatric population, with an incidence of 2.2–2.6 per 100,000 females annually (1, 2). Although ovarian neoplasms in children share histologic types with adults, their frequency distribution differs markedly: epithelial tumors predominate in adults, whereas germ cell tumors account for 60%–80% of cases in children (3, 4). Despite the low overall incidence of ovarian malignancy in this population, accurate risk stratification is essential to balance oncologic safety with fertility preservation (5–7). The diagnostic approach is particularly challenging due to a lower pretest probability of malignancy compared with adults and age-specific histopathological heterogeneity (4, 8).

To standardize the assessment of adnexal masses, the Ovarian-Adnexal Reporting and Data System (O-RADS) ultrasound system (9) was developed, providing a standardized lexicon and risk stratification tool with demonstrated excellent diagnostic performance and inter-rater reliability in adults (10–13). Despite its established efficacy in this population, the application of O-RADS in pediatric and adolescent cohorts remains less well-defined, as existing studies are often limited by small sample sizes or broad age ranges that fail to account for the significant physiological and pathological heterogeneity across childhood and adolescence (14–16). Histopathological features and hormonal status change continuously from school age to middle adolescence. These differences may substantially alter O-RADS diagnostic performance. Adult-based O-RADS risk thresholds and positive predictive value (PPV) values cannot be directly applied to pediatric populations due to their lower malignancy prevalence and distinct disease spectra.

This study aimed to analyze 501 pediatric adnexal masses and to evaluate the age-stratified performance of the O-RADS ultrasound system for distinguishing benign from malignant lesions in school-age children, early adolescents, and middle adolescents, thereby providing evidence to inform the application of O-RADS in this heterogeneous population.

## Materials and methods

### Patients

The study was conducted according to the guidelines of the Declaration of Helsinki and approved by the Institutional Review Board of the Second Xiangya Hospital, Central South University. The informed consent was waived due to its retrospective nature. Between January 2015 and January 2026, a total of 4,050 pediatric patients (aged 6–17 years) who underwent gynecological ultrasound (US) examination were retrospectively evaluated.

A total of 3,549 patients were excluded as follows: (1) absence of US identified masses (*n* = 2,019), (2) without recognized reference standards (*n* = 1,323); (3) pregnancy (*n* = 2), (4) previous treatment (*n* = 9), (5) an unclear final diagnosis (*n* = 3), (6) masses that only underwent repeated US surveillance (*n* = 173), or (7) lost or poor-quality images (*n* = 20). At last, 501 pediatric patients were enrolled in our study.

### Ultrasound image acquisition and analysis

The gynecological ultrasound examinations were performed using GE Voluson E8 and E10 (GE Healthcare); Philips iU22, EPIQ 5, and EPIQ 7 (Philips Healthcare); Esaote MyLab (Esaote S.p.A.); and Mindray R7 (Mindray Healthcare) systems. Imaging parameters were adjusted by the radiologists performing the ultrasound examination. Each target mass was routinely imaged in both the transverse and long-axis planes to obtain its largest dimensions, along with one Doppler image on the long-axis plane. The radiology reports were documented using a standardized form that included the following items: location, largest diameter, composition, echogenicity, cyst type (specified as unilocular or multilocular, with or without solid tissue), Doppler pattern, and the presence or absence of acoustic shadowing, ascites, and irregular external contour. All ultrasound images were stored in the Picture Archiving and Communication Systems.

Two radiologists (Yan Tan and Rongsen Zhang, with 3 years and more than 10 years of experience in gynecological US, respectively) randomly reviewed all the US images in consensus. Both readers had not been involved in the original examinations and were blinded to the final diagnosis and other imaging findings of patients. Discrepancies were resolved through consultation with a senior expert (Minghui Liu, with more than 30 years' experience in gynecological US). In patients with two or more masses, only the largest was designated as the target. Each mass was assessed according to the O-RADS US lexicon (9).

The O-RADS classification system categorizes masses into the following groups based on malignancy risk: O-RADS 0 for incomplete evaluation; O-RADS 1 for definitely benign (normal ovaries); O-RADS 2 for almost certainly benign (<1% risk); O-RADS 3 for low-risk (1% to <10%); O-RADS 4 for intermediate-risk (10% to <50%); and O-RADS 5 for high-risk (≥50%).

To assess inter-observer agreement on O-RADS categorization between radiologists with different levels of experience, two radiologists (Yan Tan and Rongsen Zhang, with 3 and more than 10 years of experience in gynecological US, respectively) performed a separate analysis of all masses, describing the ultrasound features and classifying them according to the O-RADS lexicon.

### Reference standard

The reference standard was established through histological confirmation obtained via surgery or biopsy. Borderline tumors were included in the malignant cohort for analysis, given their equivalent surgical management. An O-RADS score of 4 was chosen as the critical cutoff; this threshold separates masses with a low risk of malignancy (considered benign) for categories ≤3 from those carrying an intermediate to high risk (considered malignant) for categories 4 and 5.

### Definition of Age groups

Patients were categorized by age into the following groups: infants (<1 year), toddlers (1–3 years), preschoolers (4–5 years), school-age children (6–9/10 years), early adolescents (10/11–14 years), and middle adolescents (15–17 years) (17).

### Statistical analysis

The Kolmogorov–Smirnov test was used to test whether quantitative variables were normally distributed. Continuous variables are expressed as median with interquartile range (IQR). The Mann–Whitney U test was used for non-normally distributed quantitative data and Chi-square test was used for qualitative data.

The diagnostic performance was assessed in terms of accuracy, sensitivity, specificity, PPV, and negative predictive value (NPV). The area under the curve (AUC) was used to estimate the probability of predicting malignant adnexal masses. The DeLong test was used to compare different AUCs.

To evaluate interreader agreement on US features and mass categorization, we employed a single-rater, absolute-agreement, two-way random-effects model to compute intraclass correlation coefficient (ICC) estimates. The level of agreement was determined based on the following ICC thresholds: < 0.20 indicated poor agreement; 0.21–0.40, fair; 0.41–0.60, moderate; 0.61–0.80, substantial; and 0.81–1.0, almost perfect (18).

Statistical analyses were performed using Statistical Package for Social Sciences (version 29.0, IBM Corp, Chicago, IL) and MedCalc software (version 15.2.2, MedCalc Software, Ostend, Belgium). *P*-values < 0.05 (2-tailed) were considered statistically significant.

## Results

### Patient characteristics and adnexal masses

A total of 501 patients with 501 adnexal masses were included. Table 1 summarizes their characteristics. The ages of the patients ranged between 6 and 17 years. Median lesion size was 7.2 cm (IQR, 5.0–9.7) in school-age children, 8.1 cm (IQR, 5.8–13.9) in early adolescents, and 7.7 cm (IQR, 5.8–11.1) in middle adolescents. Most lesions were benign across all groups, with the highest rate in middle adolescents (93.6%), followed by school-age children (86.1%) and early adolescents (85.3%). Malignant lesions were least common in middle adolescents (6.4%) compared to 13.9% and 14.7% in the other groups.

**Table 1:** Clinical and ultrasound characteristics of adnexal masses in school-age children, early adolescents, and middle adolescents.

Characteristics	School-age children group (*n* = 72)	Early adolescents group (*n* = 163)	Middle adolescents group (*n* = 266)
Lesion diameter* (cm)	7.2 (5.0–9.7)	8.1 (5.8–13.9)	7.7 (5.8–11.1)
Location			
Left adnexa	28 (38.9)	79 (48.5)	132 (49.6)
Right adnexa	44 (61.1)	84 (51.5)	134 (50.4)
Final diagnosis			
Benign	62 (86.1)	139 (85.3)	249 (93.6)
Malignant	10 (13.9)	24 (14.7)	17 (6.4)
Lesion category			
Unilocular cyst without solid tissue	46 (63.9)	106 (65.0)	190 (71.4)
Multilocular cyst without solid tissue	14 (19.4)	27 (16.6)	45 (16.9)
Unilocular cyst with solid tissue	2 (2.8)	1 (0.6)	3 (1.1)
Multilocular cyst with solid tissue	2 (2.8)	9 (5.5)	7 (2.6)
Solid	8 (11.1)	20 (12.3)	21 (8.0)
Color score			
1	58 (80.5)	119 (73.0)	208 (78.2)
2	10 (13.9)	25 (15.3)	46 (17.3)
3	4 (5.6)	11 (6.8)	7 (2.6)
4	0	8 (4.9)	5 (1.9)
Ascites	0	11 (6.8)	4 (1.5)
Acoustic shadow	0	3 (1.8)	5 (1.9)
Irregular external contour	8 (11.1)	26 (16.0)	28 (10.5)

*Data are the median, with the range in parentheses. Except where indicated, data are numbers of lesions and numbers in parentheses are percentages.

### Histopathological types by age group

 Table 2 presents the distribution of histopathological types of adnexal masses across school-age children (*n* = 72), early adolescents (*n* = 163), and middle adolescents (*n* = 266). Benign lesions predominated in all groups, with mature teratoma being the most common subtype, followed by serous/mucinous cystadenoma and ovarian/embryonic residual cysts. Malignant tumors were most frequent in early adolescents (*n* = 24), with immature teratoma and yolk sac tumor being the leading malignant subtypes. Borderline cystadenoma was exclusively observed in adolescent groups.

**Table 2:** Histopathological types of adnexal masses in school age children, early adolescents, and middle adolescents.

Histopathological types	School age children (*n* = 72)	Early adolescents (*n* = 163)	Middle adolescents (*n* = 266)
Benign	62	139	249
Mature teratoma	49 (79.0)	67 (48.2)	103 (41.4)
Serous cystadenoma and mucinous cystadenoma	8 (12.9)	32 (23.0)	80 (32.1)
Ovarian cyst and embryonic residual cyst	4 (6.5)	38 (27.4)	47 (18.9)
Ovarian endometrial cyst	0	1 (0.7)	8 (3.2)
Adnexal inflammatory mass	0	0	2 (0.8)
Benign sex cord-stromal tumor		1 (0.7)	9 (3.6)
Vascular malformation	1 (1.6)	0	0
Malignant	10	24	17
Immature teratoma	4 (40.0)	6 (25.0)	4 (23.5)
Yolk sac tumor	2 (20.0)	6 (25.0)	2 (11.8)
Dysgerminoma	2 (20.0)	3 (12.5)	1 (5.9)
Mixed germ cell tumor	2 (20.0)	3 (12.5)	1 (5.9)
Malignant sex cord-stromal tumor	0	3 (12.5)	2 (11.8)
Mucinous cystadenocarcinoma	0	0	1 (5.9)
Borderline cystadenoma	0	3 (12.5)	6 (35.2)

### O-RADS categories by age and malignancy risk

**Figure 1:**
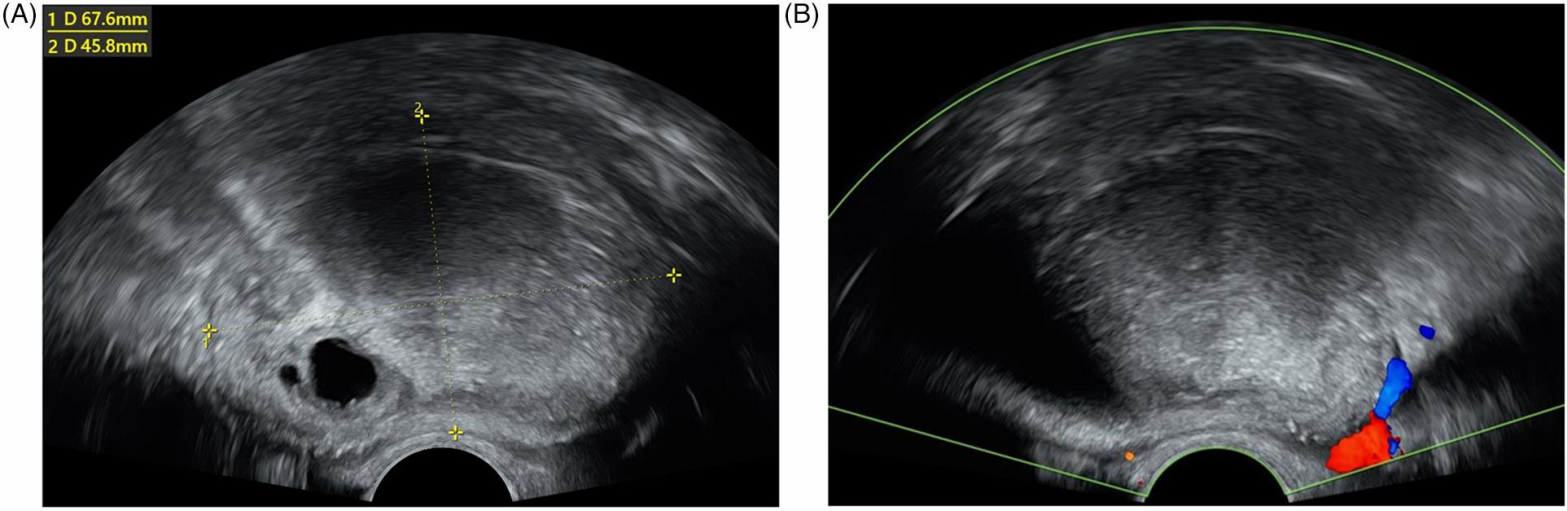
Ultrasound images from a 17-year-old girl with a confirmed mature teratoma. **(A)** Conventional ultrasound image shows a classic benign lesion with a diameter of 6.8 cm (calibers). **(B)** Color Doppler image shows no vascularity (Color score = 1). The mass was classified as O-RADS 2.

**Figure 2:**
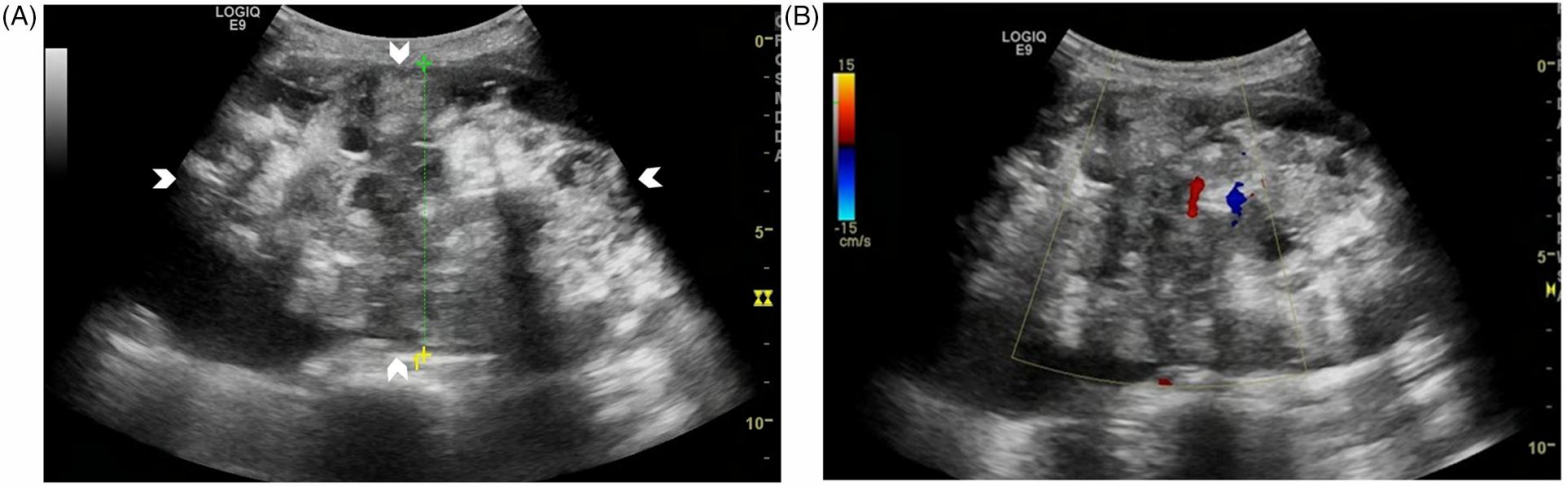
Ultrasound images from a 12-year-old girl with a confirmed immature teratoma. **(A)** Conventional ultrasound image shows a 13.9 cm irregular outer contour solid lesion (arrowheads). **(B)** Color Doppler image shows minimal vascularity (Color score = 2). The mass was classified as O-RADS 5.

**Figure 3:**
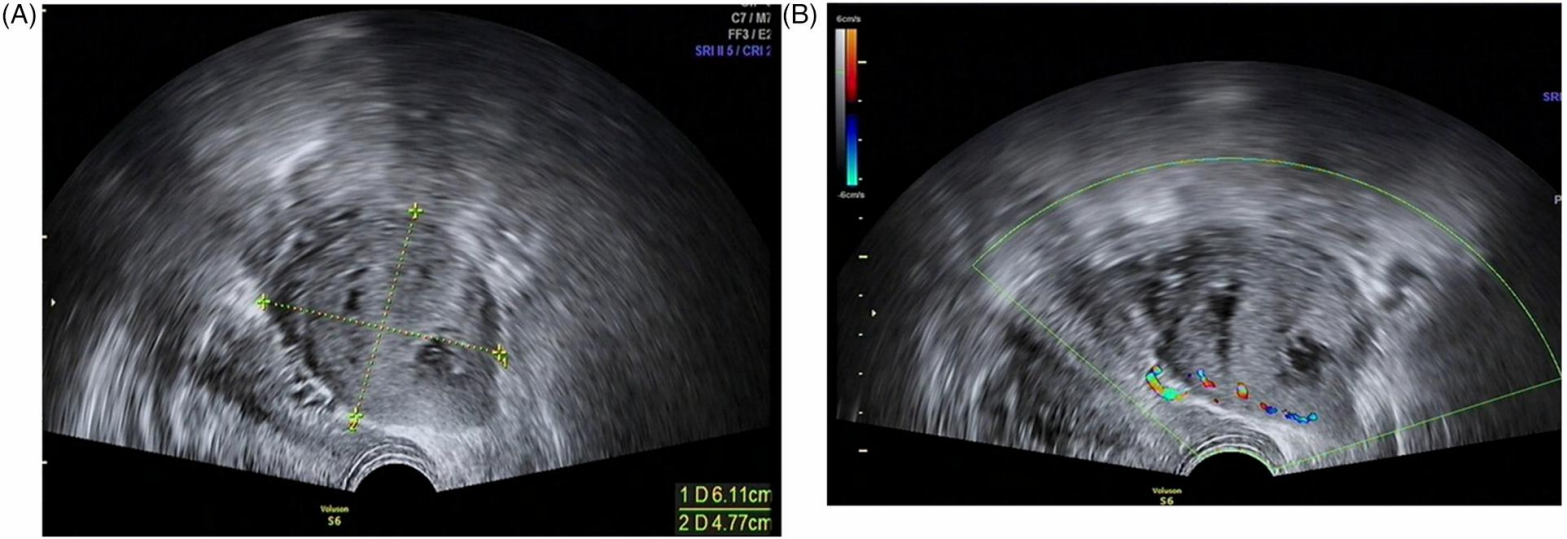
Ultrasound images from a 15-year-old girl with a confirmed ovarian sclerosing stromal tumor. **(A)** Conventional ultrasound image shows a 6.1 cm smooth outer contour solid lesion with non-shadowing artifact (calibers). **(B)** Color Doppler image shows minimal vascularity (Color score = 2). The mass was classified as O-RADS 4.

**Figure 4:**
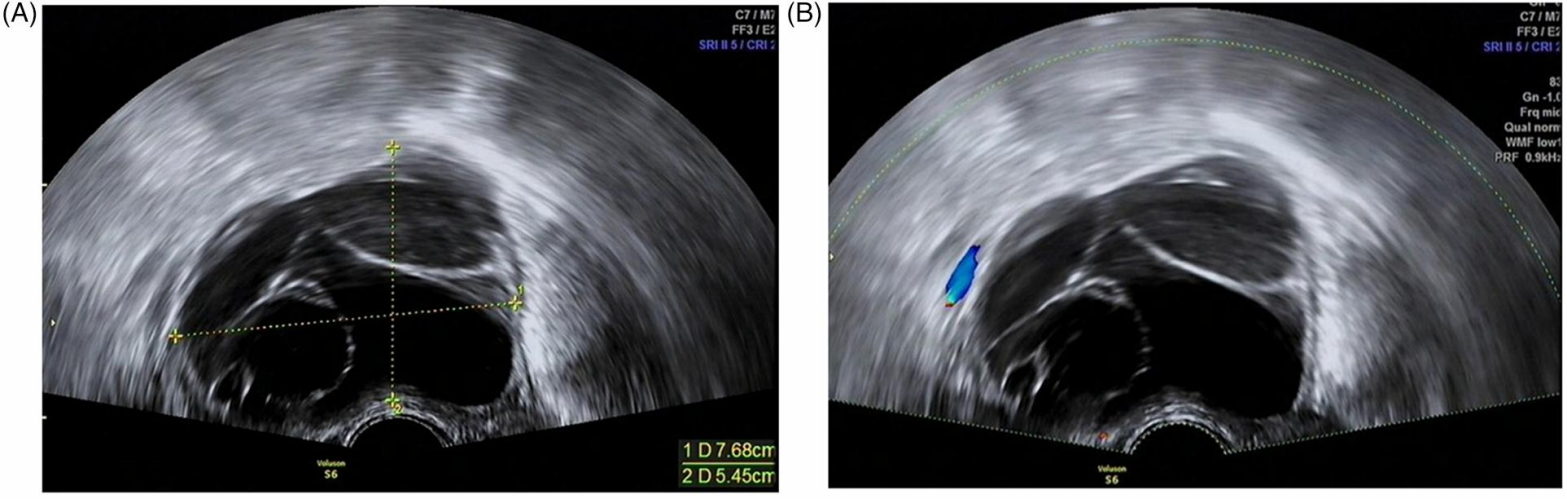
Ultrasound images from a 16-year-old girl with a confirmed mucinous cystadenoma. **(A)** Conventional ultrasound image shows a 7.7 cm multilocular cyst with smooth inner walls and septations (calibers). **(B)** Color Doppler image shows no vascularity (Color score = 1). The mass was classified as O-RADS 3.

The distribution of O-RADS categories across age groups is summarized in Table 3. All O-RADS 2 category masses are benign lesions (Figure 1). Among school-age children, all malignant lesions (*n* = 10) were classified as O-RADS 4 (40.0%) or 5 (60.0%), while no malignancies were observed in O-RADS 2 or 3. In early adolescents, the majority of malignancies (83.3%) fell into O-RADS 5 (Figure 2), with the remaining cases distributed across O-RADS 3 (4.2%) and 4 (12.5%). Similarly, in middle adolescents, 52.9% of malignant lesions were categorized as O-RADS 5, followed by O-RADS 4 (35.3%) (Figure 3) and O-RADS 3 (Figure 4).

**Table 3:** Distribution of O-RADS categories and malignancy risk in school age children, early adolescents, and middle adolescents.

O-RADS	Suggested ROM in Adults (%)	School age children (*n* = 72)		Early adolescents (*n* = 163)		Middle adolescents (*n* = 266)	
		Benign (*n* = 62)	Malignant (*n* = 10)	Benign (*n* = 139)	Malignant (*n* = 24)	Benign (*n* = 249)	Malignant (*n* = 17)
2	<1	38 (61.3)	0	75 (54.0)	0	134 (53.8)	0
3	1–10	20 (32.3)	0	48 (34.5)	1 (4.2)	79 (31.7)	2 (11.8)
4	10–50	4 (6.4)	4 (40.0)	14 (10.1)	3 (12.5)	33 (13.3)	6 (35.3)
5	≥50	0	6 (60.0)	2 (1.4)	20 (83.3)	3 (1.2)	9 (52.9)

*O-RADS*, ovarian-adnexal reporting and data system, *ROM* risk of malignancy.

Except where indicated, data are numbers of lesions and numbers in parentheses are percentages.

A total of 56 false-positive and 3 false-negative cases were identified. Among the 56 false-positive cases, the most common pathological subtypes were serous/mucinous cystadenomas (*n* = 32, 57.1%), mature teratomas (*n* = 12, 21.4%), benign sex cord-stromal tumors (*n* = 8, 14.3%), ovarian/embryonic residual cysts (*n* = 2, 3.6%), and adnexal inflammatory masses (*n* = 2, 3.6%). Among the 3 false-negative cases, two were borderline tumors and one was a yolk sac tumor.

### O-RADS performance in different age groups

The diagnostic performance of O-RADS across the three age groups is summarized in Table 4. In school-age children, the sensitivity and NPV were both 100.0%, with a specificity of 93.6% and an AUC of 0.968 (95% CI: 0.897–0.995). In early adolescents, the sensitivity was 95.8%, specificity 88.5%, and AUC 0.922 (95% CI: 0.869–0.958). In middle adolescents, the sensitivity decreased to 88.2%, specificity to 85.6%, and AUC to 0.869 (95% CI: 0.822–0.907). The PPV showed a marked decline with increasing age, from 71.4% in school-age children to 59.0% in early adolescents and 29.4% in middle adolescents. Comparative analysis of AUCs revealed a significant difference between middle adolescents and school-age children (*P* = 0.027), while no significant differences were observed between early adolescents and school-age children (*P* = 0.118) or between middle adolescents and early adolescents (*P* = 0.276). We further analyzed the diagnostic performance of O-RADS according to pathological subtypes, and the detailed results are shown in Supplementary Table S1.

**Table 4:** Diagnostic performance of O-RADS in identifying malignant pediatric adnexal lesions Among school Age children, early adolescents, and middle adolescents.

Age Group	Sensitivity (%)	Specificity (%)	PPV (%)	NPV (%)	Accuracy (%)	AUC	*p*-value*
School-age children	100.0 (69.2–100.0)	93.6 (84.3–98.2)	71.4 (49.2–86.6)	100.0	94.4	0.968 (0.897–0.995)	-
Early adolescents	95.8 (78.9–99.9)	88.5 (82.0–93.3)	59.0 (47.4–69.7)	99.2 (84.7–99.9)	90.0	0.922 (0.869–0.958)	0.118
Middle adolescents	88.2 (63.6–98.5)	85.6 (80.6–89.7)	29.4 (22.7–37.1)	99.1 (96.7–99.7)	85.7	0.869 (0.822–0.907)	0.027, 0.276

*PPV*, positive predictive; *NPV,* negative predictive value; *AU,* area under the curve.

Data in parentheses are 95% confidence intervals.

* Only AUCs were compared. The first *P*-value was compared with school-age children, the second *P*-value was compared with early adolescents.

To further evaluate whether combining additional clinical and sonographic features could improve diagnostic accuracy beyond O-RADS alone, we constructed a multivariable logistic regression model incorporating age, lesion size, color Doppler score, solid components, ascites, acoustic shadowing, and irregular external contour. Tumor markers were not included due to a high proportion of missing data in this retrospective cohort.

Multivariable analysis identified solid components (OR = 51.61, 95% CI: 12.85–207.26, *P* < 0.001), color Doppler score (OR = 10.67, 95% CI: 2.17–52.54, *P* = 0.004), and irregular external contour (OR = 4.44, 95% CI: 1.41–14.05, *P* = 0.011) as independent predictors of malignancy. Age also showed independent predictive value (OR = 0.41, 95% CI: 0.19–0.88, *P* = 0.023) (Supplementary Table 2). The multivariable model demonstrated excellent diagnostic performance with an AUC of 0.976 (95% CI: 0.959–0.988), which was significantly higher than that of O-RADS alone (AUC=0.908, 95% CI: 0.878–0.933; *P* = 0.012). This suggests that combining multiple sonographic features with age may improve risk stratification compared with O-RADS alone in the overall cohort.

Sensitivity analysis using O-RADS thresholds of 3, 4, and 5 showed that the optimal balance was achieved at a cutoff of ≥4 (AUC=0.908, sensitivity 94.1%, specificity 87.6%). A lower cutoff (≥3) increased sensitivity to 100% but substantially reduced specificity to 54.9%, while a higher cutoff (5) improved specificity to 98.9% but markedly decreased sensitivity to 68.6% (Supplementary Table S3).

### Inter-observer agreement

The inter-observer agreement for the O-RADS category was almost perfect (0.87; 95% CI: 0.85, 0.89; *P* < 0.001).With respect to the description of ultrasound features, we found almost perfect inter-observer agreement for the identification of ascites (0.88; 95% CI: 0.86, 0.90; *P* < 0.001), classification of mass categories (0.87; 95% CI: 0.84, 0.89; *P* < 0.001), and external contour (0.95; 95% CI: 0.94, 0.96; *P* < 0.001). The inter-observer agreement for color scores was substantial (0.75; 95% CI: 0.70, 0.78; *P* < 0.001).

## Discussion

The present study demonstrated that the O-RADS ultrasound system exhibits favorable diagnostic performance in school-age children, with sensitivity and NPV reaching 100.0% and specificity of 93.6%, and in early adolescents, with sensitivity of 95.8% and NPV of 99.2%, with AUC values exceeding 0.92 in both groups. However, its performance declined notably in middle adolescents, reflected by a significant reduction in AUC (0.869) and a marked drop in PPV (29.4%). These findings underscore the importance of age-specific considerations when applying O-RADS risk stratification in pediatric populations.

The favorable diagnostic performance observed in school-age children is noteworthy. In this group, all malignant lesions were correctly classified as O-RADS 4 or 5, yielding 100% sensitivity and NPV, which is consistent with the findings of Zhang et al. (15). This high accuracy may be attributed to the distinct histopathological profile of this age group. Malignant masses in school-age children were predominantly immature teratomas and mixed germ cell tumors. These entities typically present with characteristic ultrasound features, including large solid components, marked vascularity, and heterogeneous echotexture (19, 20), which align closely with the high-risk descriptors defined in the O-RADS lexicon (9). Moreover, the lower prevalence of benign mimics in this group may reduce diagnostic ambiguity, contributing to the high specificity of 93.6%.

In contrast, the diagnostic performance of O-RADS in middle adolescents showed a significant decline, particularly in PPV and AUC. Several factors may account for this observation. First, the histopathological spectrum in middle adolescents shifts toward a predominance of benign mature teratomas and functional cysts, which together constituted over 60% of lesions in this group. These benign entities often exhibit ultrasound features that overlap with intermediate-risk or high-risk categories, including complex cystic morphology, mural nodules, or mild internal vascularity. Consequently, they are often over-classified as O-RADS 4 or 5 (11, 13). Second, the prevalence of malignancy in middle adolescents was notably lower than in school-age children and early adolescents. Given that PPV is directly influenced by disease prevalence (21), the lower pretest probability in this age group inevitably reduces the positive predictive value, even when test specificity remains relatively high.

The notable difference in AUC between middle adolescents and school-age children suggests that O-RADS performance may vary across pediatric age groups. Interestingly, no significant differences were observed between early adolescents and the other two groups, which may imply that early adolescence represents a transitional phase, one in which diagnostic performance remains robust but may begin to show signs of attenuation. Another important finding is the consistently high NPV across all age groups, indicating that O-RADS is highly reliable in ruling out malignancy when a mass is classified as O-RADS 2 or 3. This is clinically valuable, as it supports a conservative, surveillance-based approach in low-risk lesions, thereby avoiding unnecessary surgical intervention in young patients where fertility preservation is a priority (5).

We also compared our findings with three recent studies on O-RADS for pediatric adnexal masses. All these studies supported the good reliability and diagnostic efficacy of O-RADS in pediatric patients, which was consistent with our results (14–16). However, they adopted general pediatric grouping without detailed age stratification. By dividing participants into school-age children, early and middle adolescents, we further demonstrated that O-RADS performance gradually declined with age. Middle adolescents presented obviously reduced AUC and a remarkable drop in PPV, which could be explained by overlapping imaging features of benign lesions and low malignancy prevalence, as previously described by Epstein et al. (16). Our stratified analysis complements existing literature and highlights the necessity of age-adapted interpretation when applying O-RADS in children and adolescents.

In our study, menarchal status and functional cysts could not be directly assessed due to the retrospective absence of menstrual history and systematic follow-up. However, using age as a proxy, we observed that the decline in O-RADS specificity and PPV in middle adolescents coincided with the expected post-menarchal state. False-positive cases in this group were predominantly hemorrhagic corpus luteum cysts and benign sex cord-stromal tumors, which sonographically mimic O-RADS 3 or 4 features. Prospective studies incorporating menstrual history are needed to validate these findings. The age-dependent decline in O-RADS diagnostic performance is largely attributable to pubertal physiological changes, menarchal status, and functional ovarian cysts. Prepubertal school-age children have a stable endocrine status and few functional cysts; their malignancies are predominantly germ cell tumors with typical sonographic features that align well with O-RADS 4–5 criteria, yielding high sensitivity and AUC. In contrast, post-menarcheal middle adolescents experience cyclic hormonal fluctuations and active ovarian changes. Functional cysts in this group often exhibit complex morphology, mural nodules, and mild vascularity, leading to frequent overclassification as O-RADS 4 or higher, increased false-positives, and reduced specificity and PPV. These findings suggest that pubertal development and menarche substantially interfere with O-RADS risk stratification, underscoring the need for age- and puberty-adapted interpretation in pediatric populations.

This study has several limitations. First, it is retrospective and single-institutional, which may introduce selection bias. Second, the reference standard was predominantly surgical histology, which may overrepresent cases with concerning imaging features, potentially affecting estimates of specificity and PPV. Third, the relatively small number of malignant cases in school-age children limits the precision of performance estimates in this subgroup. Fourth, we did not incorporate clinical variables such as tumor markers (e.g., alpha-fetoprotein, human chorionic gonadotropin), which are routinely used in pediatric risk stratification; future studies integrating clinical and imaging data may further refine diagnostic accuracy.

In conclusion, the O-RADS ultrasound system performs well in school-age children and early adolescents but shows reduced efficacy, particularly in positive predictive value, in middle adolescents. These findings suggest that age-adapted interpretation may be valuable, but prospective multi-center studies are needed to confirm the observed age-related differences.

## Data Availability

The raw data supporting the conclusions of this article will be made available by the authors, without undue reservation.

## References

[1] Skinner MA Schlatter MG Heifetz SA Grosfeld JL. Ovarian neoplasms in children. Arch Surg. (1993) 128:849-853; discussion 853-844. 10.1001/archsurg.1993.01420200023004 8343057

[2] Taskinen S Fagerholm R Lohi J Taskinen M. Pediatric ovarian neoplastic tumors: incidence, age at presentation, tumor markers and outcome. Acta Obstet Gynecol Scand. (2015) 94:425–9. 10.1111/aogs.12598 25640522

[3] Heo SH Kim JW Shin SS Jeong SI Lim HS Choi YD, et al. Review of ovarian tumors in children and adolescents: radiologic-pathologic correlation. Radiographics (2014) 34:2039–55. 10.1148/rg.347130144 25384300

[4] Wessman S Nistér M Kokaraki G Pal N Tettamanti G Petta TB. A comprehensive population-based study of malignant ovarian tumors, including histologic and immunohistochemical review, in children and adolescents 0–19 years old in Sweden between 1970 and 2014. Gynecol Oncol. (2024) 184:206–13. 10.1016/j.ygyno.2024.01.035 38340646

[5] Minneci PC Bergus KC Lutz C Aldrink J Bence C Breech L. Reducing unnecessary oophorectomies for benign ovarian neoplasms in pediatric patients. Jama. (2023) 330:1247–54. 10.1001/jama.2023.17183 37787794 PMC10548301

[6] Stanković ZB Sedlecky K Savić D Lukač BJ Mažibrada I Perovic S. Ovarian preservation from tumors and torsions in girls: prospective diagnostic study. J Pediatr Adolesc Gynecol. (2017) 30:405–12. 10.1016/j.jpag.2017.01.008 28137453

[7] Hermans AJ Kluivers KB Wijnen MH Bulten J Massuger LF Coppus SF. Diagnosis and treatment of adnexal masses in children and adolescents. Obstet Gynecol. (2015) 125:611–5. 10.1097/aog.0000000000000665 25730223

[8] Birbas E Kanavos T Gkrozou F Skentou C Daniilidis A Vatopoulou A. Ovarian masses in children and adolescents: a review of the literature with emphasis on the diagnostic approach. Children (Basel). (2023) 10. 10.3390/children10071114 37508611 PMC10377960

[9] Andreotti RF Timmerman D Strachowski LM Froyman W Benacerraf BR Bennett GL. O-RADS US risk stratification and management system: a consensus guideline from the ACR ovarian-adnexal reporting and data system committee. Radiology. (2020) 294:168–85. 10.1148/radiol.2019191150 31687921

[10] Hack K Gandhi N Bouchard-Fortier G Chawla TP Ferguson SE Li S. External validation of O-RADS US risk stratification and management system. Radiology. (2022) 304:114–20. 10.1148/radiol.211868 35438559

[11] Almalki YE Basha MAA Nada MG Metwally MI Libda YI Ebaid NY. Ovarian-Adnexal imaging-reporting and data system (O-RADS) ultrasound version 2019: a prospective validation and comparison to updated version (v2022) in pathologically confirmed adnexal masses. Eur Radiol. (2025) 35:3080–95. 10.1007/s00330-024-11235-z 39604652

[12] Perez M Meseguer A Vara J Vilches JC Brunel I Lozano M. GI-RADS versus O-RADS in the differential diagnosis of adnexal masses: a systematic review and head-to-head meta-analysis. Ultrasonography. (2024) 43:438–47. 10.14366/usg.24105 39415417 PMC11532524

[13] Yi YY Li C Zhu WJ Hou YL. Diagnostic performance of contrast-enhanced ultrasound (CEUS) combined with ovarian-adnexal reporting and data system (O-RADS) ultrasound risk stratification for adnexal masses: a systematic review and meta-analysis. Clin Radiol. (2024) 79:e1167–75. 10.1016/j.crad.2024.05.021 38942707

[14] Wang H Wang L An S Ma Q Tu Y Shang N. American College of radiology ovarian-adnexal reporting and data system ultrasound (O-RADS): diagnostic performance and inter-reviewer agreement for ovarian masses in children. Front Pediatr. (2023) 11:1091735. 10.3389/fped.2023.1091735 36969276 PMC10030612

[15] Wu M Huang L Chen Y Wang Y Zhang M Cao J. Diagnostic accuracy of ovarian-adnexal reporting and data system, IOTA simple rules and pediatric risk of malignancy Index for pediatric adnexal lesions: comparative study. Ultrasound Obstet Gynecol. (2025) 66:361–7. 10.1002/uog.29291 40643593

[16] Epstein K Dillman J Abu Ata N Coley B Li Y Pitt S. Assessment of ultrasound ovarian-adnexal reporting & data system (O-RADS) for pediatric patients. Pediatr Radiol. (2026) 56:856–66. 10.1007/s00247-026-06546-w 41697338 PMC13035678

[17] Jaramillo-Calle DA Martinez YA Balwani M Fernandez C Toro M. Porphyria attacks in prepubertal children and adolescents. Mol Genet Metab. (2021) 133:242–9. 10.1016/j.ymgme.2021.04.008 34083144

[18] Landis JR Koch GG. The measurement of observer agreement for categorical data. Biometrics. (1977) 33:159–74. 10.2307/2529310 843571

[19] Hanafy AK Mujtaba B Yedururi S Jensen CT Sanchez R Austin MT. Imaging in pediatric ovarian tumors. Abdom Radiol (NY). (2020) 45:520–36. 10.1007/s00261-019-02316-5 31745573

[20] Sorge I Hirsch FW. [Ovarian masses in infants and children]. Radiologie (Heidelb). (2024) 64:26–34. 10.1007/s00117-023-01233-5 37947867

[21] Altman DG Bland JM. Diagnostic tests 2: predictive values. Br Med J. (1994) 309:102. 10.1136/bmj.309.6947.102 8038641 PMC2540558

