# Supplementary material for: Age-stratified diagnostic performance of the ovarian-adnexal reporting and data system for adnexal masses: a focus on school-age children, early, and middle adolescents
Source: Front Pediatr. 2026 Jul 15;14:1867445. doi: 10.3389/fped.2026.1867445 (PMC13416446; doi:10.3389/fped.2026.1867445)
Supplement: Supplementary file 1 [file Table1.docx]

**Supplementary Table S1** Diagnostic performance of O-RADS for different pathological subtypes

| pathological subtype | True positive | False positive | True negative | False negative | Sensitivity  (%) | Specificity  (%) |
| --- | --- | --- | --- | --- | --- | --- |
| mature teratoma (n=219) | - | 12 | 207 | - | - | 94.5 |
| immature teratoma (n=14) | 14 | - | - | 0 | 100 | - |
| yolk sac tumor (n=10) | 9 | - | - | 1 | 90 | - |
| Dysgerminoma (n=6) | 6 | - | - | - | 100 | - |
| Malignant sex cord-stromal tumors (n=5) | 5 | - | - | 0 | 100 | - |
| Benign sex cord-stromal tumors (n=10) |  | 8 | 2 |  | - | 20 |
| borderline tumors (n=9) | 7 | 0 | 0 | 2 | 77.8 | - |
