# Supplementary material for: Age-stratified diagnostic performance of the ovarian-adnexal reporting and data system for adnexal masses: a focus on school-age children, early, and middle adolescents
Source: Front Pediatr. 2026 Jul 15;14:1867445. doi: 10.3389/fped.2026.1867445 (PMC13416446; doi:10.3389/fped.2026.1867445)
Supplement: Supplementary file 2 [file Table2.docx]

**Supplementary Table S2** Univariate and multivariate logistic regression analysis to predict malignancy in pediatric and adolescent adnexal masses

| Parameter | Univariate Analysis | | Multivariable Analysis | |
| --- | --- | --- | --- | --- |
|  | OR (95% CI) | *P* | OR (95% CI) | *P* |
| Age | 0.61 (0.42-0.89) | 0.010 | 0.41 (0.19-0.88) | *0.023* |
| Lesion size | 4.32 (2.35-7.95) | <0.001 | 1.30 (0.47-3.65) | *0.614* |
| Color Doppler score | 89.88 (27.22-296.81) | <0.001 | 10.67 (2.17-52.54) | *0.004* |
| Solid components | 272 (79.17-934.45) | <0.001 | 51.61 (12.85-207.26) | <0.001 |
| Ascites | 45.85 (12.41-169.37) | <0.001 | 1.03 (0.22-4.81) | 0.970 |
| Acoustic shadow | -- | 0.999 |  |  |
| Irregular external contour | 18.66 (9.61-36.23) | <0.001 | 4.44 (1.41-14.05) | 0.011 |
