# Supplementary material for: Age-stratified diagnostic performance of the ovarian-adnexal reporting and data system for adnexal masses: a focus on school-age children, early, and middle adolescents
Source: Front Pediatr. 2026 Jul 15;14:1867445. doi: 10.3389/fped.2026.1867445 (PMC13416446; doi:10.3389/fped.2026.1867445)
Supplement: Supplementary file 3 [file Table3.docx]

**Supplementary Table S3** Sensitivity analyses using different positive thresholds for O-RADS

| Cutoff | Sensitivity  (%) | Specificity  (%) | PPV  (%) | NPV  (%) | AUC |
| --- | --- | --- | --- | --- | --- |
| 3 | 100.0  (93.0 - 100.0) | 54.9  (50.2 - 59.6) | 20.1  (18.5 - 21.8) | 100.0  (-) | 0.774  (0.735 - 0.810) |
| 4 | 94.1  (83.8 - 98.8) | 87.6  (84.1 - 90.5) | 46.2  (39.9 - 52.5) | 99.2  (97.8 - 99.7) | 0.908  (0.880 - 0.932) |
| 5 | 68.6  (54.1 - 80.9) | 98.9  (97.4 - 99.6) | 87.5  (74.2 - 94.5) | 96.5  (94.9 - 97.7) | 0.838  (0.802 - 0.869) |
| *PPV* positive predictive, *NPV* negative predictive value, *AUC* area under the curve  Data in parentheses are 95% confidence intervals | | | | | |
